# Education of Caregivers and Veterans to Improve the Care of the Geriatric Psychiatric Patient

**DOI:** 10.1093/geroni/igab046.1906

**Published:** 2021-12-17

**Authors:** Kristen Sorocco

**Affiliations:** OKC VA Health Care System, Oklahoma City, Oklahoma, United States

## Abstract

The geriatric psychiatry outpatient clinic provides assessment of the elderly Veteran with mental illness and behavioral and psychological symptoms of dementia. I will describe strategies developed and implemented in this setting to provide education to the caregiver (family) to improve early identification of delirium, depression and cognitive impairment. This education proved to reduce the number of pharmacological treatment and increase the use of nonpharmacological interventions based on "what matters to the patient" and following the BEERS criteria guidelines. One of the most important outcomes of the education and evaluation in the geriatric psychiatric clinic was a decrease in number of emergency room visits of elderly, specifically those with dementia.

